# The role of MELK in cancer: mechanisms and therapeutic options

**DOI:** 10.3389/fonc.2026.1938187

**Published:** 2026-09-18

**Authors:** Kritika Jangir, Prachi Patidar, Bhavika Baweja, Prerna Vats, Chainsee Saini, Niranjan Kumar, Rashi Soni, Ashok Kumar, Rajeev Nema

**Affiliations:** 1 Department of Biosciences, Manipal University Jaipur, Jaipur, Rajasthan, India; 2 Department of Biochemistry, All India Institute of Medical Sciences (AIIMS), Bhopal, Bhopal, Madhya Pradesh, India

**Keywords:** cancer stemness, EMT, MELK, mitotic kinase, therapeutics

## Abstract

Cells undergo tight regulation during the mitotic phase of the cell cycle, which produces genetically identical daughter cells and prevents the segregation of duplicate chromosomes. This regulatory network has identified maternal embryonic leucine zipper kinase (MELK) as a proliferation-associated regulator. MELK is highly expressed in actively dividing cells and is often overexpressed in multiple human cancers. Beyond its canonical role in mitotic regulation, MELK is implicated in numerous oncogenic processes, including transcriptional and epigenetic regulation, inflammation, epithelial-mesenchymal transition (EMT), DNA damage response (DDR), and treatment resistance. Even though pharmacological inhibition of MELK has shown encouraging anticancer effects in preclinical models, its biological requirement remains context-dependent and poorly understood. By integrating mechanistic insights with translational and clinical evidence, this review offers a comprehensive perspective on MELK that stands out in the existing literature for its critical evaluation of context-dependent functions and therapeutic relevance. We highlight key gaps that must be addressed to establish MELK as a reliable cancer biomarker and therapeutic target.

## 1 Introduction

Protein kinases are central regulators of cellular signaling networks that govern proliferation, metabolism, stress responses, and cell survival. Through tightly controlled phosphorylation events, they coordinate essential processes such as cell-cycle progression and adaptation to environmental cues (1, 2). Within this regulatory landscape, the AMP-activated protein kinase (AMPK) family comprises evolutionarily conserved serine/threonine kinases that function as key mediators of cellular energy homeostasis and stress adaptation (3). Beyond these canonical metabolic roles, AMPK-related kinases also contribute to the regulation of cell-cycle progression and proliferative signaling networks (4). MELK is a member of the AMPK-related kinase family and is increasingly recognized for its role in regulating cellular proliferation and mitotic progression. Consistent with this function, its expression is tightly controlled in a cell-cycle-dependent manner, with transcript and protein levels rising during S phase and peaking at the G2/M transition (5, 6). This proliferative expression pattern underscores the close association of MELK with mitotic regulatory networks and suggests its role in coordinating cell division (7).

Functionally, MELK has been implicated in regulating mitotic progression and transcriptional programs during the G2/M phase of the cell cycle (8). Its expression is driven by E2F1-dependent transcription and coordinated with G2/M regulatory networks involving Forkhead box protein M1 (FOXM1) (9). MELK has been reported to activate FOXM1 through phosphorylation, thereby promoting the expression of key mitotic regulators, including Polo-like kinase 1 (PLK1), Cyclin B1, and Aurora B, to support efficient mitotic progression (9, 10). In addition, MELK undergoes dynamic subcellular redistribution during mitosis, localizing to the cytoplasm during interphase and accumulating at the cell cortex during anaphase and telophase, suggesting a role in coordinating late mitotic events such as cytokinesis (11). Beyond its canonical role in mitotic regulation, accumulating evidence indicates that MELK participates in a broader spectrum of oncogenic processes, including epigenetic regulation (12), inflammatory signaling (13), cellular stress responses (14), survival pathways (15, 16), maintenance of cancer stem-like cells (17), metastasis (18), and therapeutic resistance. MELK dysregulation and overexpression are frequently observed across multiple human cancers, including breast cancer (19), glioblastoma (12), lung cancer (20), and colorectal cancer, where its expression correlates with high mitotic activity and poor clinical outcomes. These observations suggest that MELK may function as an integrative signaling node linking proliferative signaling with tumor cell plasticity and adaptive responses.

Despite extensive investigation, the precise biological significance of MELK remains the subject of active debate. Genetic and pharmacological studies have yielded variable results, raising questions about whether MELK functions as a critical oncogenic driver or serves as a proliferation-associated marker (21). In particular, discrepancies between RNA interference-based studies and Clustered Regularly Interspaced Short Palindromic Repeats/CRISPR-associated protein 9 (CRISPR/Cas9)-mediated knockout models, along with limitations in inhibitor specificity, have complicated the interpretation of MELK-dependent phenotypes (21). These challenges highlight a critical need to re-evaluate the function of MELK within a context-dependent framework. This review therefore examines MELK biology from a context-dependent perspective, integrating its molecular architecture and regulatory networks with its roles in cell-cycle regulation, stress responses, tumor progression, and therapeutic targeting. Particular emphasis is placed on the conflicting evidence surrounding MELK dependency, the selectivity of available inhibitors, and emerging therapeutic strategies, with the aim of clarifying its potential as a biomarker and therapeutic target in cancer.

## 2 Evolution and structure of MELK

MELK is a conserved serine/threonine kinase in the SNF1/AMPK-related kinase family and is widely recognized for its association with proliferative cellular states (22). Understanding the evolutionary origin and structural organization of MELK is important for interpreting how its kinase activity and regulatory functions are controlled in both normal physiology and disease conditions. In this section, we discuss the evolutionary conservation of MELK across species and examine its key structural features, including the catalytic kinase domain and regulatory regions.

### 2.1 Evolutionary and developmental origins of MELK

MELK was initially identified in 1997 during investigations into genes involved in early mouse embryonic development (23). Using differential cDNA library analysis of unfertilized oocytes and preimplantation embryos, a novel maternally expressed transcript encoding a protein kinase was isolated. This kinase contained a serine/threonine catalytic domain with strong homology to members of the SNF1/AMPK kinase family, as well as a leucine zipper motif embedded within the catalytic domain (24). The discovery of MELK as a maternally expressed kinase, together with its independent identification as MPK38 in proliferative murine tissues, immediately suggested a functional association with rapidly dividing cell populations (25). Early expression analyses showed enrichment in hematopoietic organs, including the thymus and spleen, supporting its role in growth-associated cellular contexts (26). Furthermore, subsequent studies in *Xenopus laevis* identified the MELK homolog Eg3, characterized as a serine/threonine kinase containing the conserved catalytic subdomains typical of protein kinases and sharing strong sequence similarity with members of the KIN1/PAR-1/MARK kinase lineage, suggesting that its core biochemical functions have been evolutionarily preserved to support fundamental aspects of cell division and developmental regulation (27). Although structurally related to canonical AMPK-related kinases, MELK exhibits a distinct activation mechanism. Unlike canonical AMPK-related kinases, its activation is independent of LKB1 and instead relies on intrinsic autophosphorylation within its activation segment, thereby promoting MELK activation (28).

Functional analyses across model organisms provide further insight into the conserved biological roles of MELK kinase, as mentioned in **Table 1**. In *Xenopus laevis*, the MELK homolog Eg3 displays peak activity during mitosis and localizes to the equatorial cortex, where it is required for proper cytokinesis. Perturbation of Eg3 disrupts cleavage furrow progression without affecting mitotic entry, indicating that MELK-family kinases primarily regulate the completion of cell division rather than its initiation (7). Similarly, in *Caenorhabditis elegans*, the MELK ortholog PIG-1 governs asymmetric neuroblast division, influencing daughter cell size and fate determination (30). Loss of PIG-1 results in symmetric divisions and altered developmental outcomes, highlighting a conserved role for MELK-family kinases in polarity-dependent mitotic processes (32). The regulation of PIG-1 by the PAR-4/LKB1 pathway further links MELK-related kinases to evolutionarily conserved signaling networks that coordinate cell polarity with division.

**Table 1:** Evolutionary roles of MELK across species.

Species	MELK ortholog	Function	References
Mouse (*Mus-musculus*)	MELK	Early embryonic development	(26, 29)
Frog (*Xenopus laevis*)	Eg3	Mitotic progression and cytokinesis	(27)
Nematode (*Caenorhabditis elegans*)	PIG-1	Asymmetric cell division and fate determination	(30)
Zebrafish (*Danio rerio*)	MELK-like kinase	Hematopoietic progenitor expansion	(31)
Mammals	MELK	Lineage-restricted proliferation	(29)

In vertebrate systems, MELK function appears to be closely tied to lineage-restricted proliferation. In zebrafish, depletion of MELK-like kinases impaired primitive hematopoiesis without significantly affecting early embryonic patterning, suggesting that MELK selectively regulates proliferative expansion within specific progenitor compartments (31). Mammalian studies have demonstrated that MELK expression is spatially and temporally restricted during development, being enriched in germ cells and actively proliferating tissues while largely absent from differentiated somatic cells (29). Together, these observations highlight the evolutionary conservation of MELK function across species and underscore the importance of understanding its molecular architecture to elucidate the mechanisms regulating its kinase activity and cellular functions.

### 2.2 Structural organization of MELK

The human MELK protein consists of 651 amino acids with an approximate molecular weight of ~70–75 kDa and exhibits a modular domain organization characteristic of AMPK-related kinases. Its structure comprises an N-terminal kinase domain, followed by a ubiquitin-associated (UBA) domain and a C-terminal regulatory domain. At the N-terminus, MELK contains a catalytic kinase domain spanning approximately residues 11-263, responsible for ATP binding and substrate phosphorylation. Structural analyses indicate that this catalytic region adopts the classical bilobed architecture typical of protein kinases, in which a β-sheet-rich N-terminal lobe interacts with a predominantly α-helical C-terminal lobe to form the ATP-binding cleft that mediates substrate phosphorylation (33, 34). Immediately following the catalytic region is a UBA domain, located between residues 282 and 321, which contributes to the regulation of kinase activity. Structural studies have shown that the UBA domain forms an intramolecular interface with the N-lobe of the kinase domain, helping stabilize the catalytic conformation and influencing its activation (35). Downstream of the UBA domain, MELK contains a threonine-proline (TP)-rich regulatory region (residues ~326-530), which forms part of the extended C-terminal region and is enriched in phosphorylation sites and regulatory motifs (36). At the extreme C-terminus, MELK harbors a kinase-associated 1 (KA1) domain (residues ~550-651) that mediates protein-protein interactions and membrane association, thereby contributing to the spatial regulation of MELK signaling complexes (11), consistent with its modular domain organization and domain-specific regulatory architecture, as shown in **Figure 1**.

**Figure 1:**
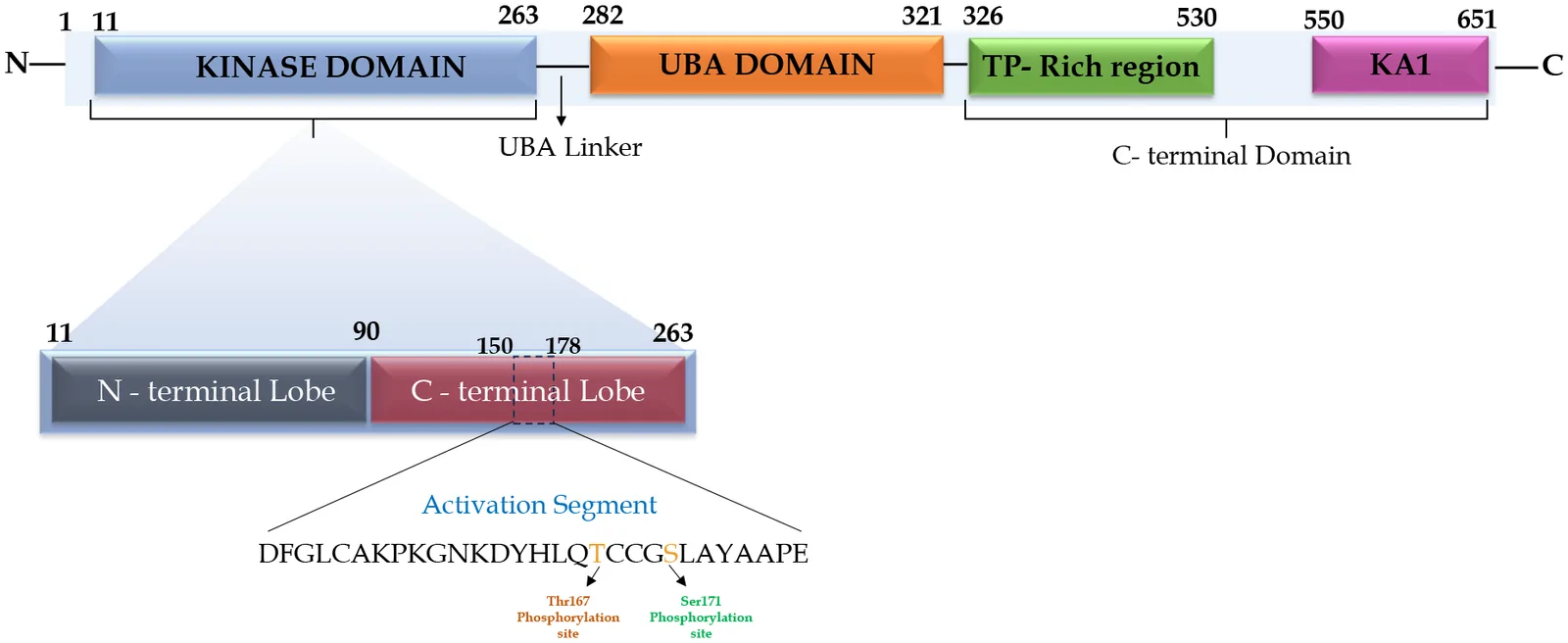
Domain organization and structural features of MELK kinase. MELK consists of the N-terminal region (residues 11-263), which comprises the serine/threonine kinase domain, which is organized into a bilobal structure consisting of an N-terminal lobe (residues ~11-90) and a C-terminal lobe (residues ~90-263). The activation segment, located within the kinase domain (approximately residues 150–178), contains key regulatory phosphorylation sites, including Thr167 and Ser171, which are critical for kinase activation. A linker region connects the kinase domain to the UBA domain (residues ~282–321), which is implicated in protein–protein interactions and regulatory functions. The C-terminal region includes a TP -rich region (residues ~326-530), followed by the KA1 domain (residues ~550-651), which is involved in subcellular localization and autoinhibitory regulation.

Additionally, MELK catalytic activity is primarily regulated by phosphorylation within the kinase domain, particularly in the activation loop. Biochemical analyses have identified its key autophosphorylation sites, including the Thr167 and Ser171 residues, which are essential for stabilizing the active kinase conformation and enabling efficient phosphorylation of downstream substrates (28). Structural evidence further indicates that phosphorylation within this region induces conformational rearrangements that align catalytic residues and facilitate ATP-dependent phospho-transfer (34). Beyond intrinsic regulation, MELK activity is further modulated by protein-protein interactions involving its C-terminal region. Thioredoxin (Trx) functions as a negative regulator, suppressing MELK kinase activity under basal conditions, whereas the zinc-finger protein ZPR9 acts as a positive regulator by enhancing interactions with downstream substrates such as apoptosis signal-regulating kinase 1 (ASK1), Smad proteins, and p53 (37, 38). Together, MELK exhibits a structural framework that enables integration of upstream regulatory cues with downstream pathways. Understanding this structural organization is critical for defining MELK regulatory mechanisms and for guiding the development of selective therapeutic inhibitors.

## 3 Multifaceted roles of MELK: from localization to signaling

MELK functions as a cell-cycle synchronized kinase that integrates proliferative signaling with mitotic execution and is consistently enriched in highly dividing cell populations and aggressive cancers (39). Initially characterized as a regulator of mitotic progression, particularly during the G2/M transition, MELK is now recognized as a multifunctional signaling node that extends beyond canonical cell-cycle control. Emerging evidence demonstrates that MELK coordinates diverse biological processes, including mitotic regulation, transcriptional and epigenetic modification, cellular stress responses, and tumor cell survival.

### 3.1 MELK localization and spatiotemporal regulation

MELK expression is tightly restricted to proliferative cellular states and is largely absent in quiescent or terminally differentiated tissues, underscoring its close association with cell division (40). During embryonic development, MELK is predominantly expressed in rapidly dividing cell populations, a pattern that is conserved across species and recapitulated in cancers with high proliferative indices (7, 41). At the molecular level, MELK expression is dynamically coordinated with cell-cycle progression. Its transcription is initiated during the late G1 phase following mitogen-induced activation of receptor tyrosine kinase (RTK) signaling and subsequent Cyclin D-CDK4/6-mediated phosphorylation of retinoblastoma (Rb) proteins, which releases E2F transcription factors to drive MELK expression through late G1 and S phases (6, 42). As cells progress toward G2, MELK transcription is further amplified by the G2/M transcriptional network, particularly through FOXM1, resulting in peak expression at the G2/M transition followed by a decline upon mitotic exit (43, 44). This tightly regulated transcriptional program ensures that MELK activity remains synchronized with proliferative demand, as shown in **Figures 2A, B**.

**Figure 2:**
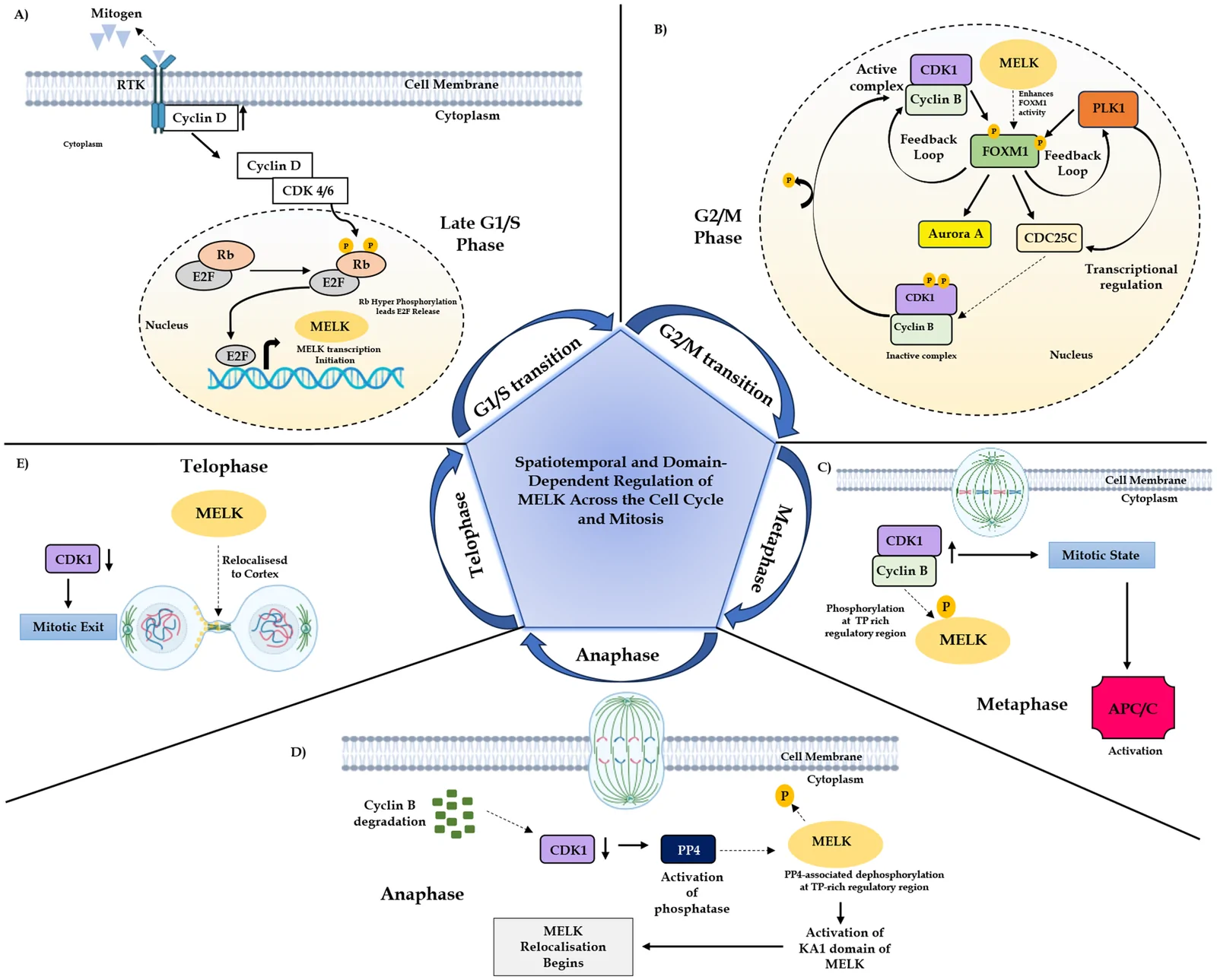
Spatiotemporal and domain-dependent regulation of MELK across the cell cycle and mitosis. **(A)** G1/S transition: Mitogenic signaling through receptor tyrosine kinases (RTKs) induces Cyclin D expression, leading to activation of the Cyclin D-CDK4/6 complex. This promotes retinoblastoma protein (Rb) phosphorylation and release of E2F transcription factors, which subsequently promote MELK transcription, linking upstream mitogenic signaling to MELK expression during the G1/S transition. **(B)** MELK participates in mitotic entry through its association with the CDK1–Cyclin B complex and FOXM1 activity. Activation of CDK1-Cyclin B initiates mitotic signaling, while MELK enhances FOXM1 activity. FOXM1 transcriptionally regulates key mitotic regulators, including PLK1, Aurora A, and CDC25C. PLK1 further promotes FOXM1 activation, while CDC25C promotes CDK1 activation by removing inhibitory phosphorylations, supporting sustained CDK1 activity and progression into mitosis. **(C)** Metaphase: Active CDK1–Cyclin B maintains the mitotic state and phosphorylates MELK at threonine-proline (TP) motifs, modulating its activity. MELK contributes to mitotic progression, while activation of the anaphase-promoting complex/cyclosome (APC/C) promotes Cyclin B degradation and prepares the cell for anaphase. **(D)** Anaphase: APC/C-mediated Cyclin B degradation reduces CDK1 activity. PP4-mediated dephosphorylation of MELK at TP sites is associated with activation of its KA1 domain and initiation of MELK relocalization, contributing to progression toward mitotic exit. **(E)** Telophase: MELK relocalizes to the cell cortex during late mitosis, coinciding with reduced CDK1 activity and completion of mitotic exit. This spatial redistribution may contribute to cytokinesis-associated processes.

In addition to transcriptional control, MELK function is governed by pronounced spatiotemporal regulation during mitosis. Live-cell imaging studies demonstrated that MELK undergoes dynamic relocalization from the cytoplasm to the cell cortex, particularly at the cleavage furrow, following the metaphase-anaphase transition, where it remains enriched until late telophase (45). This redistribution is mediated by phosphorylation-dependent intramolecular regulation. During prometaphase, elevated Cyclin-dependent kinase 1 (CDK1) activity phosphorylates residues within the threonine-proline-rich regulatory region, preventing membrane association, as illustrated in **Figure 2C**. Upon anaphase onset, declining CDK1 activity, potentially coupled with phosphatase activity such as PP4, permits the C-terminal KA1 domain to interact with membrane phospholipids, enabling cortical localization (11), as given in **Figures 2D, E**. Disruption of the KA1 domain abolished this targeting, highlighting its essential role in spatial regulation.

Functionally, this transient cortical localization suggests a role for MELK in late mitotic events, including cytoskeletal reorganization and cytokinesis, rather than a direct involvement in the spindle assembly checkpoint (SAC) pathway. This phase coincides with the activation of the anaphase-promoting complex/cyclosome (APC/C), which promotes mitotic progression by degrading Cyclin B and facilitating the transition from metaphase to anaphase. Consistently, MELK activity is further regulated by cell-cycle-dependent protein stability: it becomes phosphorylated and stabilized during mitosis and undergoes partial degradation upon mitotic exit (43). Collectively, MELK contributes to multiple regulatory layers that ensure precise temporal and spatial control of cell division.

### 3.2 MELK in regulation of cell cycle progression

Functionally, MELK contributes to cell cycle progression, particularly at the G2/M transition, where it integrates transcriptional and signaling networks that promote mitotic entry. A central mechanism involves regulation of FOXM1 activity, at least in part through phosphorylation-dependent processes, thereby influencing the transcriptional program governing G2/M progression (46). MELK has been reported to form a functional complex with FOXM1 and contribute to its phosphorylation-dependent regulation, thereby enhancing FOXM1 transcriptional activity. Notably, during the G2 phase, FOXM1 activation is initiated by CDK1-mediated priming phosphorylation at residues such as Thr596 or Ser678, thereby generating docking sites for the polo-box domain (PBD) of PLK1 (47, 48). This facilitates subsequent PLK1 binding and FOXM1 phosphorylation at Ser715 and Ser724, further enhancing its transcriptional activity (47). Within this phosphorylation cascade, MELK appears to modulate FOXM1 activity, thereby supporting the expression of downstream mitotic regulators, including PLK1 and Cyclin B1 (49). The MELK-FOXM1 axis functions within a CDK1-Cyclin B and PLK1 regulatory network. Within this framework, PLK1 promotes mitotic entry by activating the CDC25C phosphatase, which, in turn, activates the Cyclin B-CDK1 complex, forming a well-established positive feedback loop, as shown in **Figure 2B**. In addition to this canonical pathway, MELK has been reported to phosphorylate CDC25B, suggesting a potential complementary role in modulating the Cyclin B-CDK1 axis during G2/M transition (43). These findings support a role for MELK as a modulator of mitotic transcriptional programming, coordinating upstream regulatory signals with downstream kinase activation to facilitate efficient and coordinated mitotic progression, as illustrated in **Figure 3B**. Rather than functioning as a classical checkpoint kinase, MELK appears to support proliferative signaling networks that are particularly active in rapidly dividing cells.

**Figure 3:**
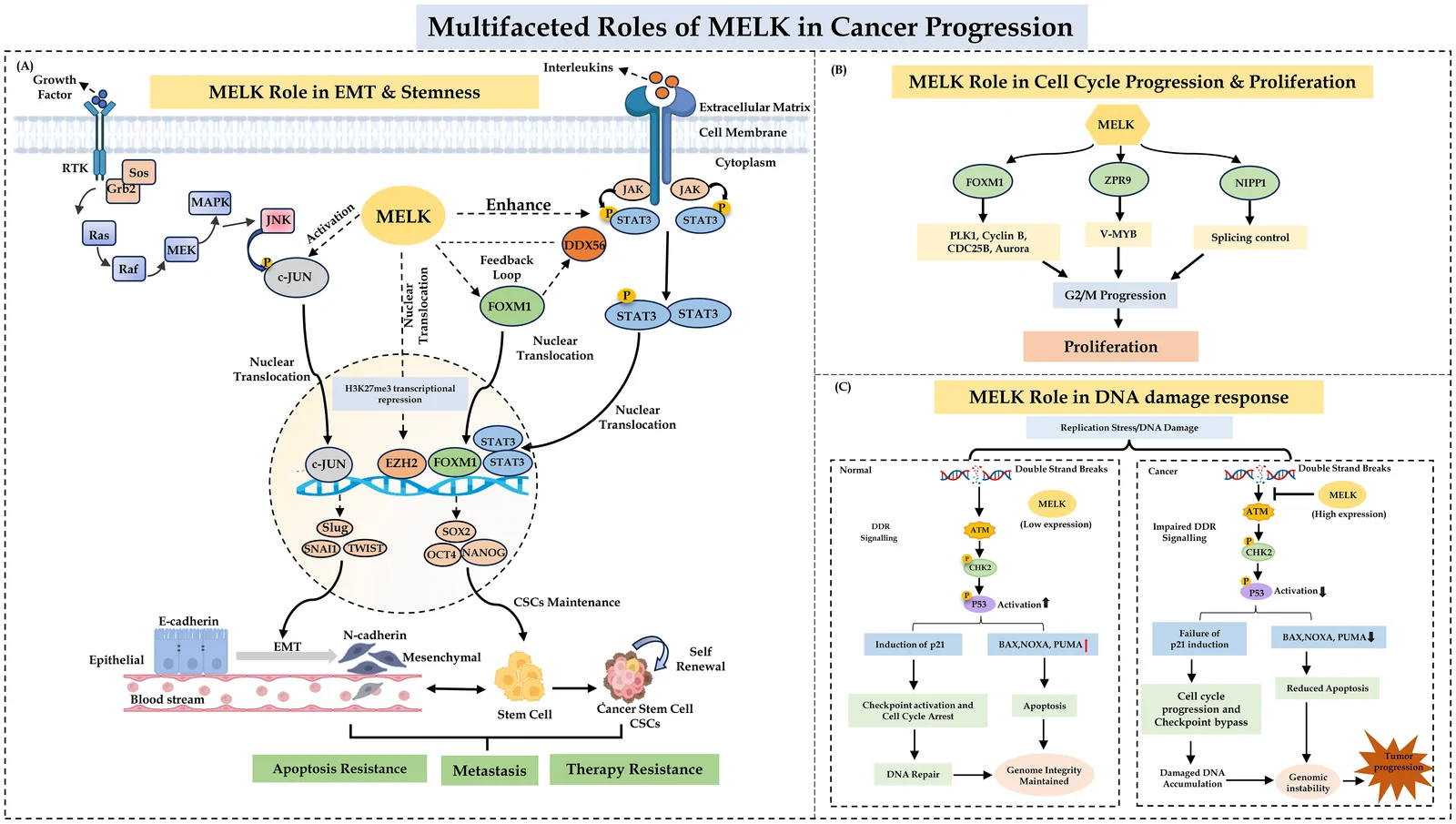
Multifaceted roles of MELK in cancer progression. **(A)** MELK in EMT and stemness: Growth factor signaling through RTKs activates the RAS-RAF-MEK-MAPK cascade and JNK/c-JUN axis, contributing to MELK activation. MELK functionally interacts with transcriptional regulators, including c-JUN, FOXM1, and DDX5, forming regulatory feedback loops, and further enhances STAT3 phosphorylation to promote its transcriptional activity. Additionally, MELK is associated with epigenetic modulation via EZH2-mediated H3K27me3. These coordinated signals promote transcription of EMT-inducing factors (*SNAI1, SLUG, TWIST*) and stemness-associated genes (*SOX2, OCT4, NANOG*). This drives EMT, maintenance of CSC, and phenotypic outcomes such as metastasis, apoptosis resistance, and therapy resistance. **(B)** MELK in cell cycle progression and proliferation: MELK regulates mitotic progression through interaction with key transcriptional and regulatory proteins. MELK enhances FOXM1 activity, leading to the transcription of mitotic regulators including PLK1, Cyclin B, CDC25B, and Aurora kinases. In parallel, MELK-associated factors, such as ZPR9, contribute to MYB-dependent transcription activation, while NIPP1 regulates splicing. These coordinated processes promote G2/M transition and sustain proliferative signaling. **(C)** MELK in DDR: Under normal or low-MELK conditions, replication stress activates the canonical ATM-CHK2-p53 signaling cascade, resulting in p21 induction, cell cycle arrest, DNA repair, and apoptosis, thereby preserving genomic integrity. In contrast, elevated MELK expression in cancer attenuates DDR signaling, leading to reduced CHK2 and p53 activation, impaired p21 induction, and diminished apoptotic responses. This facilitates checkpoint bypass, DNA damage accumulation, genomic instability, and tumor progression.

### 3.3 MELK substrates and molecular targets

MELK functions as a multifunctional kinase that regulates diverse cellular processes by phosphorylating multiple downstream substrates. Rather than acting through a single linear pathway, MELK integrates several interconnected signaling modules that collectively govern cell-cycle progression, transcriptional and epigenetic regulation, stress responses, and tumor cell survival. FOXM1 is among the best-characterized MELK substrates. FOXM1, a key downstream effector of MELK in G2/M regulation (see Section 3.2), is phosphorylated by MELK, enhancing its transcriptional activity (10). MELK functionally cooperates with CDC25B to promote activation of the CDK1-Cyclin B complex, thereby facilitating G2/M transition (50). Beyond direct cell-cycle regulators, MELK also phosphorylates ZPR9, promoting its nuclear localization and transcriptional co-activation, thereby supporting proliferation-associated gene expression programs (51). Furthermore, MELK-mediated phosphorylation of NIPP1 modulates transcription and pre-mRNA splicing, ensuring proper expression of genes required for mitotic progression (36). Collectively, these substrates act in a coordinated manner to promote efficient G2/M transition and sustain tumor cell proliferation. Importantly, MELK also targets components of the translational machinery. The eukaryotic translation initiation factor eIF4B has been identified as a direct substrate of MELK, with phosphorylation occurring at Ser406 in a mitosis-dependent manner. This finding extends MELK signaling to the regulation of mRNA translation, linking kinase activity to protein synthesis during cell division (52). Additionally, MELK phosphorylates Enhancer of Zeste homolog 2 (EZH2), reinforcing epigenetic silencing programs associated with stemness (53).

Further, it regulates cellular stress responses and apoptotic signaling by modulating key stress-responsive substrates. It phosphorylates PDK1 to regulate AKT signaling (54) and stabilizes p53 through phosphorylation, promoting apoptosis and cell-cycle arrest (55). Phosphorylation of Smad proteins (Smad2, Smad3, Smad4, and Smad7) links MELK to TGF-β signaling (56), while phosphorylation of ASK1 activates JNK and p38 MAPK (Mitogen-activated protein kinase) pathways in response to oxidative stress. MELK supports tumor cell survival by activating pro-survival signaling pathways. MELK also interfaces with STAT3 signaling, contributing to survival-associated transcriptional regulation (see Section 3.5) (57). In parallel, MELK interacts with Sequestosome 1 (SQSTM1)/p62, facilitating activation of Nuclear factor kappa B (NF-Kb) signaling and downstream survival pathways (15). These coordinated mechanisms enable tumor cells to sustain survival signaling and adapt to stress conditions. Interaction with Bcl-G further implicates MELK in apoptotic regulation (Jung et al., 2008). Upstream, MELK activity is enhanced during mitosis through phosphorylation by MPF/CDK1 and MAPK, reinforcing its role in M-phase progression (5). Collectively, MELK coordinates cell-cycle progression, transcriptional and epigenetic regulation, and stress-response signaling by phosphorylating diverse downstream substrates, as listed in **Table 2**.

**Table 2:** MELK associated substrates.

Protein	Cellular function	Role of MELK phosphorylation	Reference
FOXM1	G2/M transcription factor	Enhances transcription of mitotic genes (PLK1, Cyclin B1, Aurora B)	(10)
EZH2	Histone methyltransferase (PRC2)	Promotes H3K27me3-mediated epigenetic repression	(53)
ZPR9	Zinc-finger transcription regulator	Phosphorylation promotes nuclear accumulation and transcriptional activity	(51)
eIF4B	Translation initiation factor	Phosphorylation at Ser406 enhances translation initiation and promotes synthesis of pro-survival protein.	(52)
NIPP1	PP1 regulatory protein involved in RNA splicing	Regulates spliceosome assembly and RNA processing	(36)
Cdc25B	CDK phosphatase controlling G2/M transition	Associated with regulation of mitotic entry	(50)
PDK1	Upstream kinase in AKT signaling	Modulates AKT survival signaling	(54)
p53	Tumor suppressor transcription factor	Enhances p53-dependent apoptosis and cell-cycle arrest	(55)
Smad2/3/4/7	Mediators of TGF-β signaling	Regulates TGF-β signaling pathways	(56)
ASK1	MAP3K in stress signaling	Activates JNK and p38 MAPK pathways	(58)

### 3.4 MELK-EZH2 axis in epigenetic regulation

In addition to its role in mitotic transcriptional control, MELK contributes to epigenetic regulation by interacting with EZH2, the catalytic component of the Polycomb Repressive Complex 2 (PRC2), which mediates H3K27 trimethylation, a mark associated with transcriptional repression of differentiation and tumor suppressor genes (59, 60). Evidence indicates that MELK acts upstream of EZH2 to regulate its stability and activity, linking kinase signaling with chromatin regulation in cancer. Mechanistically, MELK directly interacts with and phosphorylates EZH2 at Ser220, a modification that suppresses ubiquitination at Lys222, thereby preventing proteasomal degradation and promoting nuclear accumulation of EZH2 (53). Stabilized EZH2 enhances PRC2-mediated H3K27me3 deposition, reinforcing repression of genes that restrict proliferation and promote differentiation (61). Functionally, cooperative activity between MELK and EZH2 promotes tumor cell proliferation and survival across multiple cancer models (12). Consistently, elevated MELK expression correlates with increased EZH2 stability and sustained epigenetic repression programs associated with malignant progression (62). The MELK-EZH2 axis represents a key mechanism linking mitotic kinase activity with epigenetic control, supporting tumor proliferation, stem-like cellular states, and resistance to differentiation.

### 3.5 MELK in stress response and cell fate regulation

MELK functions as a context-dependent regulator of cellular stress, balancing apoptotic and survival signaling. While it promotes apoptosis under physiological conditions, in cancer it is reprogrammed to support pro-survival pathways and tumor progression. In non-transformed and developmental contexts, MELK activates pro-apoptotic signaling pathways in response to cellular stress, as illustrated in **Figure 4A**. Under oxidative stress conditions, reactive oxygen species (ROS) directly activate ASK1, which, together with MELK-mediated phosphorylation of ASK1 at Thr838, enhances downstream JNK and p38 MAPK signaling, thereby promoting apoptosis (63, 64). Activation of this pathway induces the nuclear translocation of transcription factors such as AP-1, thereby contributing to the transcriptional activation of pro-apoptotic genes. In parallel, MELK phosphorylates p53 at Ser15, thereby stabilizing and promoting nuclear accumulation of p53 (23, 65). MELK also modulates TGF-β signaling by phosphorylating Smad proteins (Smad2 Ser245, Smad3 Ser204, Smad4 Ser343, and Smad7 Thr96) (56), promoting the formation of SMAD transcriptional complexes and their nuclear translocation. Together, these transcriptional regulators, AP-1, p53, and SMAD complexes, converge at the nuclear level to cooperatively drive expression of pro-apoptotic and growth-inhibitory genes, including BAX, BIM, PUMA, and NOXA (66). These coordinated transcriptional programs promote mitochondrial outer membrane permeabilization (MOMP), leading to cytochrome c release, apoptosome formation, and caspase activation, ultimately resulting in apoptosis.

**Figure 4:**
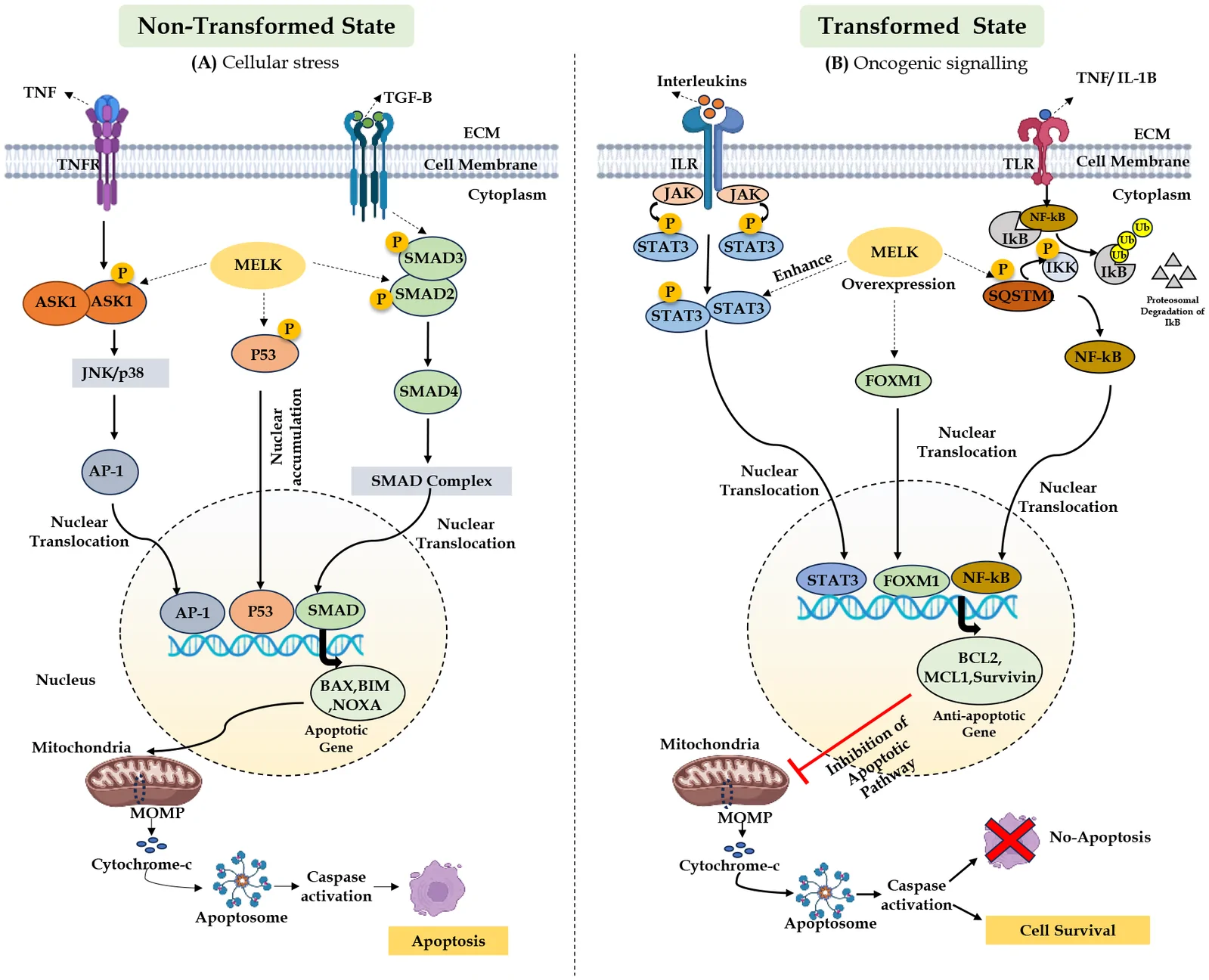
Context-dependent role of MELK in regulating stress-induced apoptosis versus cancer-associated survival signaling. **(A)** Non-Transformed state: Under stress conditions, MELK is associated with stress-responsive pathways that contribute to apoptotic signaling. TNF signaling activates ASK1, leading to JNK/p38-mediated AP-1 activation, while TGF-β induces SMAD2/3 phosphorylation and SMAD4 complex formation. Concurrently, MELK is associated with p53 activation and nuclear accumulation. These transcription factors (AP-1, p53, and SMADs) translocate to the nucleus and promote expression of pro-apoptotic genes, including BAX, BIM, and NOXA. This culminates in mitochondrial outer membrane permeabilization (MOMP), cytochrome c release, apoptosome formation, caspase activation, and execution of apoptosis. **(B)** Transformed state: In contrast, under oncogenic conditions, MELK is overexpressed and rewires signaling toward survival. Elevated MELK enhances FOXM1 activity and is associated with activation of STAT3 and NF-κB pathways through cytokine (ILR-JAK/STAT3) and inflammatory (TLR-NF-κB) signaling. MELK also interacts with SQSTM1, facilitating NF-κB activation. These transcription factors translocate to the nucleus and drive expression of anti-apoptotic genes, including *BCL2, MCL1*, and *Survivin*. This suppresses mitochondrial apoptotic signaling by inhibiting MOMP, thereby preventing caspase activation and promoting cell survival.

In contrast, MELK in cancer cells predominantly shifts toward activation of pro-survival signaling networks, as shown in **Figure 4B**. MELK overexpression promotes activation of the NF-κB pathway by phosphorylation-dependent regulation of the scaffold protein SQSTM1/p62, thereby facilitating IKK complex activation and subsequent nuclear translocation of NF-κB. This drives transcriptional programs associated with cell survival, proliferation, and inflammatory signaling (15). In parallel, MELK enhances cytokine receptor-mediated signaling via the JAK2/STAT3 pathway, in which activation of receptor-associated JAK kinases leads to phosphorylation and nuclear accumulation of STAT3, thereby promoting survival-associated transcriptional programs (57). Consistent with its role in G2/M regulation, MELK also supports FOXM1-associated transcriptional programs. Together, these transcription factors integrate pro-survival signaling programs to sustain tumor cell viability. MELK further sustains tumor cell survival by modulating metabolic and growth signaling pathways, including the AKT (Protein kinase B)/mTOR (Mechanistic target of rapamycin) axis. Phosphorylation of PDK1 contributes to downstream AKT activation, while interaction with MLST8 promotes activation of both mTORC1 and mTORC2 complexes (16). In addition to transcriptional and signaling pathways, MELK regulates tumor cell survival by modulating protein synthesis through the MELK-eIF4B axis (see Section 3.3). Phosphorylation of the translation initiation factor eIF4B enhances its activity within the translation initiation complex, promoting efficient mRNA translation during mitosis. This mechanism selectively supports the synthesis of short-lived anti-apoptotic proteins, particularly MCL1, which is essential for maintaining mitochondrial integrity and preventing apoptosis during cell division. Disruption of MELK or eIF4B function reduces MCL1 protein synthesis without significantly affecting its transcription, leading to apoptotic cell death (52). This translational control mechanism provides a critical layer of regulation, enabling MELK to sustain tumor cell survival under proliferative and mitotic stress. Beyond its tumor-cell survival functions, MELK has also been implicated in tumor microenvironment remodeling by promoting M2 macrophage polarization through the CSF-1/JAK2/STAT3 axis, thereby contributing to chemoresistance in gastric cancer (GC) (67). However, current evidence primarily supports tumor-cell-mediated effects, whereas direct functional roles of MELK in tumor-associated macrophages and other immune populations remain less clearly established. These findings indicate that the functional consequences of MELK signaling may differ according to the cellular state and tumor microenvironment, with distinct downstream pathways and phenotypes emerging in different biological contexts.

### 3.6 MELK in cancer stemness and tumor initiation

MELK has emerged as an important regulator of Cancer stem cell (CSC) biology and tumor-initiating capacity. CSC represent a subpopulation of tumor cells capable of self-renewal, tumor initiation, and sustained tumor growth (68). Consistent with this role, MELK expression is enriched in aggressive tumor populations and contributes to signaling networks that support stemness and tumor progression, as shown in **Figure 3A**. Beyond its established role in G2/M regulation (Section 3.2), the MELK–FOXM1 axis also contributes to cancer stemness. In glioma stem cells, FOXM1-dependent transcriptional programs downstream of MELK promote expression of stemness-associated factors, including SOX2, OCT4, and NANOG (10, 69). In addition to transcriptional control, MELK contributes to CSC maintenance through epigenetic mechanisms, particularly via the MELK–EZH2 epigenetic axis described in Section 3.4. Consistent with this regulatory role, disruption of either MELK or EZH2 in medulloblastoma impairs the proliferative capacity of CSC, thereby supporting the involvement of the MELK–EZH2 axis in sustaining stem-like properties (12). In this context, pharmacological inhibition of MELK suppresses the growth of glioblastoma and cancer stem-like cells by disrupting downstream signaling pathways, including AKT and FOXM1, thereby reducing proliferation and viability (17). Recent evidence further indicates that MELK integrates stemness with malignant progression pathways. The RNA helicase DDX56 promotes cancer stemness and EMT by activating the MELK-FOXM1 axis, whereas its suppression reduces FOXM1 activity and diminishes tumorigenicity in hepatocellular carcinoma (HCC) models (70). These findings position MELK as a regulator of tumor cell plasticity and stemness.

### 3.7 MELK in epithelial-mesenchymal transition and tumor invasion

EMT involves the conversion of epithelial cells into a mesenchymal-like state, characterized by reduced cell-cell adhesion and increased motility (71). MELK contributes to metastatic progression by regulating EMT and tumor cell invasion, key processes underlying cancer dissemination, as shown in **Figure 3A**. Elevated MELK expression is associated with aggressive tumor behavior across multiple cancers, and its depletion suppresses cancer cell migration and invasion (18). Mechanistically, MELK-associated signaling networks converge on transcriptional regulators that drive EMT. Activation of MAPK/JNK signaling leads to phosphorylation and nuclear translocation of c-JUN, which contributes to the transcription of EMT-associated genes (72). In parallel, MELK contributes to EMT, at least in part, through FOXM1-associated transcriptional programs (see Section 3.2), supporting the expression of EMT-related transcription factors such as Slug and Twist1, which regulate genes involved in extracellular matrix remodeling and cell motility (48, 73). These transcriptional programs promote the EMT, characterized by downregulation of epithelial markers, such as E-cadherin, and upregulation of mesenchymal markers, including N-cadherin (74). This phenotypic shift enhances cell migration, invasion, and dissemination into the bloodstream.

Beyond transcriptional regulation, MELK also modulates cytoskeletal dynamics and focal adhesion signaling. In gastric cancer, MELK regulates actin organization via RhoA-dependent signaling, promoting stress fiber and filopodia formation. Its depletion reduces these structures and decreases phosphorylation of focal adhesion kinase (FAK) and paxillin, impairing actin polymerization and cell-matrix adhesion (75). Clinically, elevated MELK expression correlates with lymph node metastasis and poor outcomes. In oral squamous cell carcinoma, MELK knockdown reduces proliferation, migration, invasion, and EMT-associated phenotypes (76). MELK promotes metastatic progression by coordinating the regulation of EMT, cytoskeletal dynamics, and focal adhesion signaling.

### 3.8 MELK in DNA damage response and regulatory networks

MELK, although not a canonical DDR kinase, has been reported to function as a context-dependent modulator of Ataxia telangiectasia mutated (ATM) mediated checkpoint signaling, as illustrated in **Figure 3C**. Under replication stress-induced DNA damage, the DDR is initiated by ATM activation, which undergoes autophosphorylation at Ser1981 and subsequently phosphorylates key downstream effectors, including CHK2 at Thr68 and p53 at Ser15. Activated p53 transcriptionally induces p21 to promote cell cycle arrest and facilitate DNA repair (77, 78), while also upregulating pro-apoptotic genes to maintain genomic integrity. In normal conditions, the ATM-CHK2-p53 axis remains intact, ensuring efficient checkpoint activation and maintenance of genomic stability. In contrast, MELK inhibition or depletion induces replication stress and DNA double-strand breaks (DSBs), accompanied by activation of the ATM–CHK2–p53 axis and subsequent p21 upregulation, indicating that MELK contributes to DNA-damage tolerance in proliferating cancer cells (79). However, the available evidence supports a functional regulatory role of MELK in the DDR signaling rather than a demonstrated direct interaction or site-specific phosphorylation of its components.

At the regulatory level, MELK expression is driven by cell-cycle associated transcription factors, particularly E2F1, linking it to proliferative signaling pathways frequently dysregulated in cancer (6). MELK expression is further modulated by post-transcriptional mechanisms, including microRNAs (miRNA), long non-coding RNAs (lncRNA), and circular RNAs (cirRNA). miRNAs such as miR-214-3p and miR-21-5p regulate MELK expression and downstream signaling pathways, while lncRNAs (e.g., PCDHB17P) and circRNAs modulate MELK through competing endogenous RNA networks, influencing pathways such as AKT/mTOR and NF-κB (80–83). These findings suggest that MELK may influence the balance between DNA repair and apoptosis, further contributing to tumor survival under therapeutic stress.

## 4 Roles of MELK across human cancers

MELK expression and functional relevance vary across tumor types, with accumulating evidence supporting a context-dependent role rather than a universal oncogenic dependency. Across multiple malignancies, MELK is consistently associated with highly proliferative and stem-like tumor states; however, its functional contribution to tumor progression varies with cellular context and tumor biology.

### 4.1 MELK in breast cancer

MELK is highly expressed in breast cancer, particularly in aggressive subtypes such as triple-negative breast cancer (TNBC), where its expression correlates with poor prognosis and increased proliferative capacity. Transcriptomic analyses consistently demonstrate elevated MELK levels in breast tumors compared with normal mammary tissue, with enrichment in highly proliferative subtypes. Clinically, increased MELK expression correlates with higher histologic grade, advanced stage, and poorer survival outcomes, particularly in TNBC, where it remains an independent prognostic factor (19). Functionally, MELK exhibits subtype-specific dependency (84). Genetic depletion selectively impairs proliferation in basal-like breast cancer cells while having minimal effects in luminal subtypes. Loss of MELK induces mitotic defects and caspase-dependent apoptosis, indicating that MELK supports cell-cycle progression in highly proliferative tumor contexts (9).

Beyond proliferation, MELK contributes to tumor progression by promoting EMT and cancer stem-like properties. MELK silencing reduced mammosphere formation, decreases CD44^+^/CD24^-^ and aldehyde dehydrogenase 1 (ALDH1)^+^ stem-like populations, and impaired migration and invasion, accompanied by reversal of EMT-associated marker expression and reduced metastatic potential *in vivo* (22). Integrated transcriptomic analyses further support these findings. MELK-high tumors are enriched for cell-cycle–associated pathways, including the G2/M checkpoint, E2F, MYC, and mitotic spindle programs, consistent with their role in proliferative signaling. These tumors also exhibit enrichment of interferon response pathways, increased infiltration of CD8^+^ T cells, M1 macrophages, and dendritic cells, and elevated cytolytic activity, indicating an association with an active immune microenvironment. Clinically, higher MELK expression correlates with increased pathological complete response (pCR) rates following neoadjuvant chemotherapy, likely reflecting the sensitivity of highly proliferative tumors to cytotoxic agents (85). Therapeutically, MELK also contributes to treatment resistance. Elevated expression is associated with reduced radiation sensitivity and increased risk of local recurrence, whereas MELK inhibition enhances radiosensitivity by impairing DNA double-strand break repair (86).

### 4.2 MELK in lung cancer

MELK is significantly upregulated in lung cancer, particularly in lung adenocarcinoma (LUAD) and small cell lung cancer (SCLC), where its expression is associated with tumor progression (87). Functionally, MELK supports the proliferation and invasion of lung cancer cells. Its depletion suppresses proliferation, migration, and invasion and induces G2/M phase arrest by disrupting the PLK1-CDC25C-CDK1 signaling axis, ultimately leading to apoptotic cell death (18). MELK also operates within non-coding RNA-mediated regulatory networks. The oncogenic lncRNA BBOX1-AS1 promotes MELK expression by sponging miR-27a-5p, thereby relieving post-transcriptional repression. Elevated MELK subsequently activates FAK signaling, driving proliferation, migration, invasion, and EMT. Both genetic silencing and pharmacological inhibition of MELK (OTSSP167) attenuate these oncogenic effects and suppress tumor growth, establishing MELK as a key functional mediator in this axis (20). Beyond its role in proliferation and invasion, MELK contributes to the maintenance of stem-like and highly proliferative states. Its inhibition induces cytokinetic defects, reduces clonogenic capacity, and suppresses sphere formation, indicating a requirement for MELK in sustaining cancer cell plasticity under proliferative stress. Mechanistically, these effects are associated with downregulation of FOXM1 and AKT-dependent signaling pathways (88).

### 4.3 MELK in gastric cancer

MELK is overexpressed in GC tissues and cell lines compared with normal gastric mucosa and is detectable in intestinal metaplasia, suggesting early involvement in tumorigenesis. Clinically, elevated MELK expression correlates with deeper tumor invasion, lymph node and distant metastasis, advanced TNM stage, and reduced overall survival and has been identified as an independent prognostic factor (89). Functionally, MELK promotes GC progression by supporting proliferation, survival, and metastatic behavior. Genetic silencing or pharmacological inhibition of MELK suppresses cell proliferation, induces G2/M arrest, and triggers apoptosis. MELK depletion also reduces migratory and invasive capacities and impairs metastatic colonization *in vivo* (89). Mechanistically, MELK facilitates EMT, characterized by decreased E-cadherin and increased expression of mesenchymal markers, and sustains survival signaling by activating the PI3K–AKT pathway. Consistent with this dependency, pharmacological inhibition of MELK using OTSSP167 suppresses tumor growth in both xenograft and patient-derived xenograft models. Genomic analyses further support MELK as a candidate oncogenic driver in gastric cancer. Recurrent amplification of the MELK locus at chromosome 9p13.3 has been observed in primary gastric tumors and across GC cell lines, providing a genomic basis for its overexpression and suggesting positive selection during tumor evolution (90).

### 4.4 MELK in colorectal cancer

Early profiling studies demonstrated that MELK is broadly expressed across colorectal cancer (CRC) cell lines and co-expressed with embryonic and CSC markers, including CD44, CD133, SOX4, and OCT4, indicating an association with stem-like tumor cell populations rather than differentiated epithelial states (91). Functional and mechanistic studies support a role for MELK in CRC progression. MELK is significantly overexpressed in CRC tissues and cell lines, and its genetic silencing suppresses proliferation, migration, and invasion. Mechanistically, MELK promotes activation of the FAK/Src-AKT signaling axis, with MELK depletion reducing AKT phosphorylation, supporting its role as an upstream regulator of pro-survival and pro-migratory signaling (92).

Additional studies revealed that MELK operates within metabolic regulatory networks. MELK functions downstream of the E2F1–xCT (SLC7A11) axis. E2F1-driven xCT expression increased MELK levels and promoted tumorigenesis and cancer stemness through AKT/mTOR signaling. Inhibition of xCT or treatment with erastin reduced MELK expression, decreases stemness-associated markers, and suppresses tumor growth and metastasis *in vivo*, supporting MELK as a downstream effector linking xCT-associated metabolic regulation to stem-like behavior (93). MELK has also been implicated in therapeutic resistance. Exposure to radiation or 5-fluorouracil increased MELK expression, while MELK knockdown sensitized CRC cells to both radiotherapy and chemotherapy by reducing proliferation and altering cell-cycle progression, supporting a role for MELK in CRC cellular responses to treatment-induced stress and resistance (94). Importantly, these findings represent the principal mechanisms directly validated in CRC, whereas other MELK-associated pathways discussed in this review have been characterized primarily in other cancer models.

### 4.5 MELK in renal cancer

MELK has been implicated in the progression of renal cell carcinoma (RCC), particularly clear cell RCC (ccRCC), through clinical and transcriptomic analyses. Large-scale the Cancer Genome Atlas (TCGA) studies identified MELK as a hub gene within survival-associated co-expression modules enriched for cell-cycle and mitotic pathways, with elevated MELK expression correlating with advanced disease stage and poor overall survival (95). Independent analyses integrating TCGA and GEO datasets further validated MELK as part of a multigene prognostic signature, in which increased expression contributes to high-risk classification and adverse clinical outcomes, and is supported by protein-level expression in tumor tissues (96). Functional and mechanistic studies demonstrated that MELK actively promotes renal cancer progression. Its expression is elevated in advanced-stage ccRCC, and its silencing suppresses proliferation, invasion, and clonogenic growth, whereas overexpression enhances malignant phenotypes. Mechanistically, MELK directly phosphorylates proline-rich AKT substrate of 40 kDa (PRAS40), relieving its inhibitory effect on mTORC1 and promoting sustained mTOR signaling activation, thereby linking MELK to metabolic growth control (97). Additional evidence indicates that MELK is a component of mitotic regulatory networks. MELK functions within a signaling axis involving T-LAK cell-originated protein kinase (TOPK) and the transcription factor FOXM1; pharmacological inhibition with OTS167 reduced cell viability, suppresses FOXM1 expression, and impairs proliferation. Combined targeting of MELK and TOPK produces additive anti-tumor effects, indicating a coordinated role in mitotic signaling pathways (98).

### 4.6 MELK in prostate cancer

MELK is significantly upregulated in prostate cancer, particularly in high-grade and aggressive disease. Expression profiling and immunohistochemical analyses demonstrated elevated MELK levels in high-grade tumors compared with benign tissue and low-grade disease, with strong correlations to Gleason score and co-expression of mitotic regulators such as ubiquitin-conjugating enzyme E2 C (UBE2C), DNA topoisomerase II alpha (TOP2A), Cyclin B2 (CCNB2), and Aurora kinase B (AURKB), linking MELK to proliferative tumor programs and poor clinical outcomes (99). Functional studies from the same work further showed that MELK depletion reduced cell viability and proliferation, supporting an active role in tumor cell survival rather than a passive association with proliferation. Cross-species analyses integrating genetically engineered mouse models and human datasets further established MELK as a driver of disease progression. MELK expression increases during tumor progression, is conserved between mouse and human prostate cancers, and is significantly associated with biochemical recurrence, reinforcing its relevance in aggressive disease (100).

Mechanistic studies have identified MELK as an upstream regulator of the transcription factor E2F8, defining a MELK-E2F8 axis in advanced prostate cancer. MELK-dependent upregulation of E2F8 promotes tumor growth and clonogenic capacity, and co-elevated expression of MELK, E2F8, and androgen receptor signaling components is associated with poor prognosis, implicating this axis in castration-resistant prostate cancer (101). Systems-level analyses using Bayesian network modeling further position MELK as a central regulator within transcriptional networks driving progression and therapeutic resistance. Pharmacological targeting with cyclosporin A suppresses E2F8 expression in a MELK-dependent manner and reduces tumor growth, suggesting therapeutic potential through disruption of MELK-driven transcriptional programs.

### 4.7 MELK in melanoma

MELK plays a functionally important role in melanoma progression and therapy resistance. It is significantly overexpressed in primary and metastatic melanoma compared with normal skin, and its expression is transcriptionally regulated by oncogenic MAPK signaling through E2F1. Functional studies demonstrated that MELK is required for melanoma cell proliferation and clonogenic growth and that its kinase activity is essential for tumor cell survival. Mechanistically, it promotes melanoma growth by activating NF-κB signaling via direct phosphorylation of SQSTM1/p62, thereby supporting downstream survival pathways activated by MAPK signaling. MELK inhibition suppresses the growth of vemurafenib-resistant melanoma cells and delays the emergence of resistance to v-Raf murine sarcoma viral oncogene homolog B (BRAF) inhibitors, highlighting its role as a bypass survival kinase in targeted therapy resistance (15). Complementing these findings, human genetic analyses identified germline variants near the MELK locus that are significantly associated with poor overall and melanoma-specific survival and confirmed elevated MELK expression in metastatic lesions, supporting its relevance as a prognostic determinant in melanoma patients (102).

### 4.8 MELK in hepatocellular carcinoma

MELK is significantly overexpressed in HCC and is associated with early recurrence and poor clinical outcomes. Analysis of paired tumor and adjacent non-tumor tissues demonstrated that its high expression correlates with reduced recurrence-free and overall survival and serves as an independent predictor of early postoperative recurrence (103). Functional studies from the same work showed that MELK knockdown suppresses proliferation, induces apoptosis, and causes mitotic arrest, accompanied by increased phospho-histone H3 levels. Additionally, MELK silencing also reduced colony formation, invasion, and tumor growth in xenograft models, as well as the expression of the stemness markers CD44 and CD133. Gene expression analyses further revealed a strong association between MELK and FOXM1-regulated mitotic and cell-cycle programs, thereby linking MELK to the proliferative signaling that underlies recurrence.

Independent transcriptomic analyses identified MELK as a hub gene within co-expression modules enriched for cell-cycle regulators, including CDK1, Cyclin B1 (CCNB1), AURKB, and TOP2A, with higher expression associated with advanced tumor stage and aggressive molecular subtypes (104). Complementary large-scale analyses confirmed its consistent overexpression associated with poor overall and disease-free survival. These studies further demonstrated that MELK dysregulation is driven by transcriptional and epigenetic mechanisms rather than copy-number amplification, including a ceRNA network involving LINC00265 and MIR3142HG-mediated suppression of miR-101-3p, as well as promoter hypomethylation (104). More recent mechanistic work revealed a metabolic role for MELK in HCC progression. It activates the PI3K/AKT/mTOR pathway to upregulate the cuproptosis-related gene dihydrolipoamide S-acetyltransferase (DLAT), thereby enhancing mitochondrial function, promoting oxidative phosphorylation, reducing oxidative stress, and supporting tumor growth and metastasis. Pharmacological induction of cuproptosis using the copper ionophore elesclomol counteracts MELK-driven tumor progression, identifying a metabolically defined therapeutic vulnerability (105).

### 4.9 MELK in brain cancer

MELK is highly expressed in brain cancers, particularly glioblastoma and medulloblastoma, where it is enriched in tumor stem-like cell populations and associated with aggressive disease behavior. In glioblastoma, MELK is selectively expressed in tumor-initiating cells but is minimally expressed in normal neural progenitors, suggesting a tumor-specific role. Functional and mechanistic analyses demonstrated that MELK is required for stem cell survival and self-renewal and directly interacts with and activates the transcription factor FOXM1, driving expression of mitotic genes essential for tumor proliferation. Disruption of the MELK–FOXM1 axis impairs glioma stem cell growth and reduced tumor formation *in vivo*, establishing MELK as a key regulator of stemness and mitotic signaling (12). Independent studies further support MELK as a tumor-selective dependency in brain cancer. Its silencing induced apoptosis and loss of self-renewal capacity, while pharmacological inhibition reduced intracranial tumor growth and prolongs survival in xenograft models (106).

MELK also integrates stress and differentiation signaling in high-grade gliomas. It is regulated by JNK-c-JUN signaling and functionally antagonizes p53, thereby promoting differentiation and suppressing tumor growth (107). Its elevated expression in recurrent and radioresistant tumors further links it to therapy resistance and disease recurrence (108). In medulloblastoma, MELK is similarly overexpressed in stem-like tumor cells and promotes proliferation and maintenance of an undifferentiated state. Mechanistically, it interacts with and phosphorylates EZH2, forming a regulatory loop that sustains epigenetic repression and tumor growth, while its inhibition induces differentiation and reduced tumorigenicity (10). Transcriptomic analyses across glioma cohorts further identify MELK as a marker of high tumor grade and poor prognosis, with expression correlating with malignant molecular features and reduced overall survival (109). A summary of these cancer-specific functions is provided in **Table 3**. Future studies integrating genetic dependency models with clinical data will be essential to define tumor contexts in which MELK represents a viable therapeutic vulnerability.

**Table 3:** MELK Pan cancer perspective.

Cancer type	MELK expression	Clinical/survival association	Cohort/data source (n)	Analysis	Evidence type	References
Breast cancer	Higher in TNBC/aggressive subtypes	Higher MELK associated with poorer OS, PFS and DMFS	World IBC Consortium; n=314	Kaplan–Meier; Cox regression	Clinical cohort	(19)
	Higher in basal-like than luminal tumors	MELK depletion impaired proliferation and survival preferentially in basal-like models	TCGA: 392 tumors, 61 normal tissues; five independent cohorts; breast cancer cell lines/xenografts	Differential-expression; functional analysis	Clinical + preclinical	(9)
	Highest in basal-like tumors; associated with copy-number gain	Expression associated with basal-like subtype and copy-number status	RNA-seq n=50 tumors; TCGA CNA n=1,075; CCLE 45 breast cancer cell lines; IHC 86 tissue cores	Differential-expression; copy-number correlation; FISH; IHC	Multi-cohort genomic/expression	(84)
	High in aggressive/stem-like tumors	Associated with poorer survival and metastatic features; depletion reduced mammosphere formation, invasion and metastasis	Public expression datasets; TNBC cell lines; *in vivo* metastasis models	Survival correlation; functional analysis	Bioinformatic + preclinical	(22)
	High in proliferative/immune-active tumors	Higher MELK associated with increased pCR after neoadjuvant chemotherapy	GSE25066 n=508; GSE20194 n=248; HESS n=133	ROC/AUC; survival/correlation analysis; immune-cell analysis	Clinical/transcriptomic	(85)
	Elevated in basal-like/TNBC tumors	Associated with radioresistance and local recurrence	TCGA + independent clinical datasets; cell lines/xenograft models	Kaplan–Meier; Cox regression; expression; clonogenic assays	Clinical + preclinical	(86)
Lung cancer	Upregulated in LUAD	Higher MELK associated with poorer OS and advanced disease	75 LUAD tumors + 35 adjacent normal tissues; TCGA/GEPIA/Oncomine/KM Plotter	Differential-expression; IHC; Kaplan–Meier	Clinical/tissue + bioinformatic	(87)
	Highly expressed in LUAD	MELK depletion reduced proliferation, migration and invasion	LUAD cell lines; xenograft models; public expression datasets	Expression; knockdown; functional; xenograft analysis	Preclinical + transcriptomic	(18)
	Upregulated; positively associated with BBOX1-AS1	MELK contributed to proliferation, migration, invasion and EMT through FAK signaling	76 NSCLC tumor/adjacent normal samples; GSE18842, GSE19188, GSE29250, GSE33532; A549/SK-MES-1	Differential-expression; correlation; Kaplan–Meier; functional analysis	Clinical/tissue + preclinical	(20)
	Highly expressed in SCLC	MELK inhibition reduced cell viability, sphere formation and tumor growth	11 SCLC cell lines; 6 primary SCLC vs 17 normal lung tissues; CCLE 53 SCLC lines; xenografts	Expression; siRNA/inhibitor; functional; xenograft analysis	Preclinical	(88)
Gastric cancer	Expressed in GC cell lines and tumors	MELK depletion/inhibition reduced proliferation, invasion, tumor growth and metastatic colonization	GC cell lines; xenografts; 3 GC-PDX models (2 MELK-positive, 1 MELK-negative)	Functional assays; xenograft/PDX analysis	Preclinical *in vitro/in vivo*	(89)
	MELK copy-number gain at 9p13.3	Copy-number gain may contribute to MELK overexpression	4 GC cell lines; 68 primary gastric adenocarcinomas; gain in 38/68 (55.9%)	Array-CGH; FISH; qPCR	Genomic + primary tumor	(90)
Colorectal cancer	MELK expressed in most CRC cell lines; associated with stem-cell markers	Association with stem-like tumor-cell phenotype	13 CRC cell lines derived from 10 primary and 3 metastatic tumors	Gene-expression profiling; marker analysis	Cell-line/expression	(91)
	Overexpressed in CRC tissues and cells	MELK knockdown reduced proliferation, migration and invasion	CRC tissues and CRC cell models; exact patient *n* not reported in accessible study information	RT-qPCR; MTT; Transwell; Western blot	Clinical/tissue + preclinical	(92)
	MELK positively associated with xCT and stemness-related signaling	MELK supported xCT-mediated tumorigenesis and stemness	TCGA: 41 COAD + 9 READ tumor/paired normal samples; GEO GSE87211/GSE17538/GSE39582; validation 49 CRC + 65 normal samples; xenograft models	RNA-seq; correlation; GSEA; functional/rescue assays; xenograft	Clinical/transcriptomic + preclinical	(93)
	MELK increased after radiation or 5-FU	MELK suppression sensitized cells to radiation and 5-FU	SNU-503 rectal cancer cell model; additional CRC cell lines for baseline expression	RT-PCR; siRNA; radiation/5-FU treatment; cell-cycle/proliferation assays	Preclinical	(94)
Renal cancer	Upregulated in advanced ccRCC; associated with mitotic pathways	Higher MELK associated with advanced disease and poorer survival	TCGA 348 samples (265 stage I, 83 stage IV); GSE73731–85 samples (41 stage I, 44 stage IV)	Differential-expression; Limma; GO/KEGG; survival analysis	Transcriptomic/prognostic	(95)
	Included in a seven-gene prognostic signature	High-risk score associated with poorer OS	TCGA-ccRCC n=526; validation datasets	WGCNA; LASSO; Kaplan–Meier; Cox; ROC/AUC	Multi-cohort transcriptomic	(96)
	Upregulated in stage IV ccRCC	MELK promoted malignant phenotype and disease progression	TCGA + GSE73731; ccRCC cell models	Differential-expression; loss/gain-of-function; functional analysis	Transcriptomic + preclinical	(97)
	Highly expressed in kidney cancer cells	MELK inhibition reduced growth; combined MELK/TOPK inhibition produced additive suppression	5 kidney cancer cell lines: VMRC-RCW, Caki-1, Caki-2, 769-P, 786-O	Correlation; siRNA; inhibitor; viability assays	Preclinical functional	(98)
Prostate cancer	Upregulated in high-grade tumors; correlated with mitotic genes	Associated with high-grade disease	Discovery microarray n=98; validation qPCR n=79; TMA n=94	Differential-expression; qPCR; IHC; correlation	Clinical	(99)
	Increased during progression; conserved between mouse and human tumors	High MELK associated with biochemical recurrence	Two genetically engineered mouse models + human prostate cancer datasets; Cambridge cohort n=111 in recurrence analysis	Cross-species transcriptomics; recurrence analysis; functional analysis	Preclinical	(100)
	Positively correlated with E2F8 and increased during progression	High E2F8/AR associated with poorer prognosis; MELK identified upstream of E2F8	TCGA-PRAD; cBioPortal; GSE3325; prostate cancer cell lines/xenografts	Correlation; Bayesian network; knockdown; functional analysis	Transcriptomic +Priclinical	(101)
Melanoma	Upregulated in BRAF-mutant melanoma through MAPK–E2F1 signaling	MELK inhibition suppressed melanoma growth and delayed BRAF-inhibitor resistance	A375, M14, SKMEL-28, SKMEL-239 and other melanoma models; xenograft models	Phosphoproteomics; knockdown/inhibitor; functional analysis	Preclinical	(15)
	MELK variant associated with expression; elevated in metastatic lesions	rs60970102 associated with poorer OS; HR = 3.14 (95% CI 2.05–4.81)	Discovery n=551; validation n=550 early-stage immunotherapy-naïve patients	GWAS; multivariable Cox; survival/transcriptomic analysis	Clinical	(102)
HCC	Overexpressed; associated with mitotic/FOXM1 programs	High MELK associated with early recurrence and poorer survival	HCC tumor/adjacent tissue cohort; HCCLM3/HuH7; xenograft models	IHC; survival; knockdown; functional; xenograft analysis	Clinical + preclinical	(103)
	Upregulated; hypomethylation associated with expression	Higher MELK associated with poorer OS; independent prognostic factor	TCGA-LIHC cohort; TCGA, Oncomine, ICGC and GEO datasets	Differential-expression; Kaplan–Meier; Cox; methylation/ceRNA analysis	Multi-cohort transcriptomic	(104)
	Upregulated; associated with advanced stage	High MELK associated with poorer outcomes; promoted tumor progression and elesclomol resistance	106 HCC tissues; 16 paired tissues for qPCR; 6 paired tissues for WB; TCGA-LIHC, ICGC-LIRI, GSE14520; cell/xenograft models	Differential-expression; Kaplan–Meier; functional; xenograft analysis	Clinical + preclinical	(105)
Glioblastoma	Highly expressed in GSCs/GBM; correlated with FOXM1	MELK–FOXM1 signaling promoted GSC growth and tumor formation	Grade III glioma n=24; GBM n=56; TCGA GBM n=218; GSC models/xenografts	Expression/correlation; functional; xenograft analysis	Transcriptomic + preclinical	(12)
	Highly expressed in GBM/GSCs	OTSSP167 reduced proliferation/self-renewal and prolonged survival	U87, U251, A172, T98G, LN229, LN18; GSC models; intracranial xenografts; n=10/group for reported U87 survival analysis	Functional; Kaplan–Meier; xenograft analysis	Preclinical	(106)
	Elevated in HGG/GSCs; increased after irradiation/recurrent disease	Higher MELK associated with shorter postsurgical survival; linked to radioresistance	Tissue microarray n=91 HGG tumors; GSC models; recurrent HGG cohort 41 primary + 30 recurrent	IHC; Kaplan–Meier; functional; radiation studies	Clinical/tissue + preclinical	(107)
	MELK/EZH2 elevated in recurrent/post-irradiation tumors	Associated with poorer prognosis and radioresistance	HGG microarray n=76; newly diagnosed GBM n=65; recurrent GBM n=41; poor/good prognosis subsets n=17/19; 3 glioma sphere models	Expression; survival correlation; radiation; functional analysis	Clinical/tissue + preclinical	(108)
	Upregulated; associated with higher WHO grade and malignant features	High MELK associated with poorer OS	CGGA n=1,018 for expression; CGGA n=636 + TCGA n=592 with clinical information; GEO validation datasets	Kaplan-Meier;	Multi-cohort transcriptomic/prognostic	(109)

## 5 Therapeutic targeting of MELK kinase

MELK has emerged as a potential therapeutic target due to its elevated expression in highly proliferative and aggressive tumors. Early studies demonstrated that pharmacological inhibition of MELK suppresses tumor cell proliferation, induces apoptosis, and reduces tumor growth in preclinical models. These findings initially positioned MELK as a promising candidate for targeted cancer therapy.

### 5.1 Direct therapeutic targeting of the MELK

#### 5.1.1 OTSSP167 (OTS167)

OTSSP167 is a first-in-class, ATP-competitive MELK inhibitor with potent nanomolar activity (IC_50_ ≈ 0.41 nM) (110). It suppresses MELK-dependent signaling and inhibits proliferation and CSC properties, accompanied by modulation of downstream regulators including FOXM1 and p53 (111). Preclinical studies demonstrated broad antitumor activity across multiple malignancies, including breast cancer (111), leukemia (112), neuroblastoma (113), ovarian (114), lung (88), kidney (98), and gastric cancers (75), where it showed effects on tumor growth, metastasis, and therapy resistance. These promising preclinical findings led to clinical evaluation of OTSSP167. However, its clinical development has remained limited. The Phase I/II study NCT02795520, which evaluated intravenous (IV) OTS167 in patients with relapsed or refractory haematological malignancies, was terminated in 2021 because of slow patient recruitment. Separately, the Phase I study NCT02926690 is evaluating oral OTS167 in advanced breast cancer, including a dose-expansion cohort for TNBC, and remains recruiting. Beyond clinical development, pharmacokinetic properties may also influence the therapeutic utility of OTSSP167, particularly in tumors requiring central nervous system penetration. Preclinical studies showed that OTSSP167 is a substrate of the efflux transporters MDR1 and BCRP at the blood-brain barrier (BBB), substantially restricting its brain penetration and bioavailability (115). Notably, interpretation of OTSSP167-mediated effects is complicated by its limited target selectivity. In addition to MELK, OTSSP167 can inhibit other kinases, including Aurora B, BUB1, and Haspin. Moreover, the persistence of antiproliferative activity following MELK knockout suggests that some effects of OTSSP167 may occur independently of MELK, raising concerns regarding its on-target specificity and complicating the interpretation of its cellular effects (116, 117). These findings highlight the need for more selective next-generation MELK inhibitors and biomarker-guided identification of MELK-dependent tumors to improve target validation and therapeutic specificity.

#### 5.1.2 MELK-8a and MELK-8b

MELK-8a and MELK-8b are structure-guided small-molecule inhibitors developed through virtual screening and medicinal chemistry optimization to target the ATP-binding pocket of the MELK kinase domain. MELK-8a exhibits potent inhibitory activity (IC_50_ ≈ 2 nM), indicating strong suppression of MELK catalytic function (118). Functionally, MELK- 8a selectively inhibited proliferation of MELK-dependent cancer cells, particularly in TNBC models, while showing minimal effects on MELK-independent cells, supporting a context-dependent reliance on MELK signaling. This activity is associated with delayed mitotic entry and a transient G2 cell-cycle arrest rather than an apoptotic response (118). Mechanistically, MELK-8a also disrupts MELK-mediated phosphorylation of SQSTM1/p62, thereby attenuating NF-κB signaling and suppressing melanoma cell proliferation and tumor growth (15). These findings indicate that MELK-8a and MELK-8b selectively target MELK-driven signaling networks and may offer greater specificity than earlier inhibitors.

#### 5.1.3 MELK-T1

MELK-T1 is an early, selective ATP-competitive inhibitor of MELK that targets the kinase catalytic domain and suppresses its phosphorylation activity, with an IC_50_ of 37nM (79). In addition to inhibiting kinase activity, MELK-T1 promoted proteasome-dependent degradation of MELK, thereby reducing its protein abundance and functional activity. Functionally, MELK-T1 induced replication stress and activates the DDR, characterized by ATM-CHK2-p53 signaling, p21 upregulation, and γH2AX accumulation, leading to replication fork stalling, S-phase arrest, and cellular senescence. Mechanistically, MELK is proposed to elevate the threshold for DNA damage checkpoint activation in proliferating cancer cells, whereas MELK-T1 lowered this threshold, restoring ATM-dependent checkpoint signaling and increasing sensitivity to replication stress. Consistent with this model, genetic depletion of MELK recapitulated these effects, supporting an on-target mechanism of action (79). MELK-T1 therefore provides pharmacological evidence linking MELK activity to replication-stress tolerance and DNA damage checkpoint regulation, although further evaluation is required to establish its therapeutic selectivity.

#### 5.1.4 CRO15

CRO15 is a biguanide-derived small molecule developed from metformin analogs and functions as a dual AMPK/MELK-targeting agent. It exhibits improved potency over metformin (IC_50_ ~2.5–5 µM) and reduced tumor cell viability in melanoma models (119). Mechanistically, CRO15 induced mitochondrial dysfunction and activated AMPK while concurrently inhibiting MELK activity, leading to suppression of proliferative signaling pathways, including NF-κB and cell-cycle regulators, and activation of the ATM-p53-REDD1 axis. These effects collectively promoted autophagy and apoptosis (119).

#### 5.1.5 HTH-01-091

HTH-01–091 is an ATP-competitive MELK inhibitor that targets the catalytic kinase domain, with an IC_50_ of 10.5 nM (120). Functionally, HTH-01–091 directly suppresses MELK catalytic activity. However, pharmacological inhibition with HTH-01–091 produced minimal effects on cellular proliferation, consistent with the limited phenotypic effects observed following genetic MELK knockout (120). However, subsequent studies demonstrated that MELK inhibition is not universally required for proliferation across all cancer models, particularly in certain basal-like breast cancers, highlighting context-dependent sensitivity to MELK targeting. As a result, HTH-01–091 is primarily utilized as a research tool to study MELK-dependent signaling rather than as a clinically advanced therapeutic agent. Overall, direct MELK inhibitors differ in their inhibitory potency, cellular selectivity and mechanisms of action, as summarized in **Table 4**.

**Table 4:** Direct inhibitor of MELK.

Inhibitor	Mode of action	IC_50_	Cell selectivity	Clinical phase	Structure
OTSSP167 (OTS167)	ATP-competitive MELK inhibitor	0.41 nM (enzymatic/cell-free kinase assay)	Inhibits MELK-overexpressed cancer cells A549 (lung), T47D (breast), DU4475 (breast), and 22Rv1 (prostate) with cellular IC50 values of 6.7, 4.3, 2.3, and 6.0 nM, respectively	Phase I clinical trials (NCT02926690, NCT02795520)	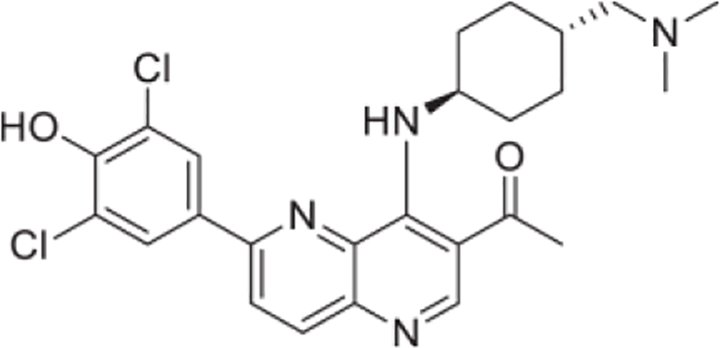
MELK-8a (NVS-MELK8a)	ATP-competitive MELK inhibitor; induces G2/M arrest via delayed Aurora A, Aurora B, CDK1 activation	2 nM	TNBC (MDA-MB-468); highly selective	Preclinical	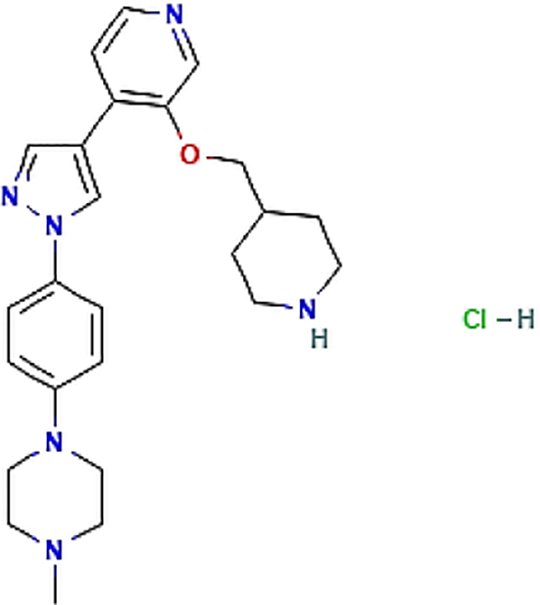
MELK-T1	ATP-competitive MELK inhibitor; also promotes proteasome-dependent MELK degradation activates ATM-CHK2-p53 DNA damage response	37nM	Induces replication stress, S-phase arrest, and senescence in MCF-7 (breast cancer) cells	Preclinical	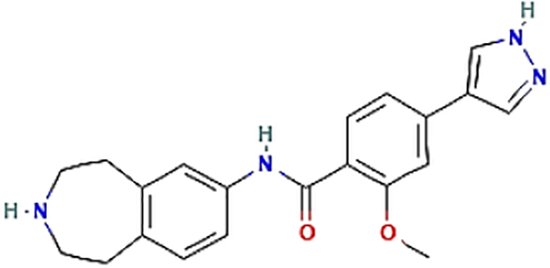
CRO15	induces mitochondrial dysfunction	2.5–5 μM	Active in melanoma models; dual AMPK/MELK activity prevents attribution of cellular effects exclusively to MELK	Preclinical	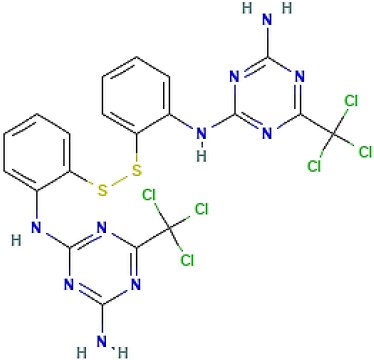
HTH-01-091	ATP-competitive MELK inhibitor targeting the catalytic kinase domain	10.5 nM	MDA-MB-468, ZR751 TNBC cell line model	Preclinical	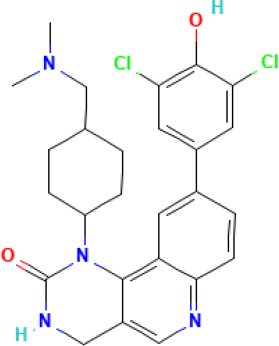

### 5.2 Direct and indirect natural inhibitors of MELK

#### 5.2.1 Corosolic acid

Corosolic acid is a pentacyclic triterpenoid that inhibits MELK–FOXM1 signaling and suppresses retinoblastoma (Y-79) cell proliferation. It induces G2/M arrest via p53/p21 activation and downregulation of mitotic regulators, while promoting mitochondrial apoptosis. Notably, it reduces both MELK and FOXM1 expression, highlighting its role as an indirect modulator of MELK-dependent transcriptional programs (121).

#### 5.2.2 Phillygenin

Phillygenin is a natural lignan compound that directly targets MELK, as supported by molecular docking and functional studies. It suppresses proliferation, migration, and invasion while promoting apoptosis in pancreatic cancer cells, and its effects are partially reversed by MELK overexpression, confirming target specificity. Additionally, phillygenin inhibits EMT by modulating E-cadherin and vimentin expression, indicating a role in limiting metastatic potential (122).

#### 5.2.3 Siomycin A and thiostrepton

Siomycin A and thiostrepton are thiazole antibiotics that indirectly target MELK signaling by inhibiting the transcription factor FOXM1, a key downstream effector of MELK. Siomycin A suppresses FOXM1-dependent target genes, including CENP-B, Cdc25B, and Survivin, inducing apoptosis in human cancer cell lines of diverse origin, while thiostrepton downregulates FOXM1-dependent mitotic regulators such as Cyclin B1, PLK1, and Aurora B, inducing G1 and S phase cell-cycle arrest and apoptosis. Through disruption of the MELK-FOXM1 axis, these agents impair mitotic progression and tumor cell survival, with demonstrated activity in breast cancer and leukemia models (123–125). Overall, natural compounds targeting MELK highlight alternative strategies to modulate MELK-driven oncogenic pathways, although their effects are largely indirect and context-dependent, as mentioned in **Table 5**.

**Table 5:** Direct and Indirect Natural Inhibitor of MELK.

Inhibitor	Target in MELK pathway	Mode of action	Reference
Siomycin A	FOXM1	Inhibits FOXM1 transcriptional activity and suppresses mitotic genes (Cyclin B1, Cdc25B, Survivin)	(123, 124)
Thiostrepton	FOXM1	Suppresses FOXM1 expression and FOXM1-dependent mitotic regulators (Cyclin B1, PLK1, Aurora B), causing G2/M arrest and apoptosis	(124, 125)
Corosolic Acid	MELK–FOXM1 axis	Natural compound that suppresses MELK and FOXM1 expression and induces p53/p21-mediated G2/M arrest and apoptosis	(121)
Phillygenin	MELK	Natural lignan that interacts with MELK and suppresses proliferation, migration, invasion, and EMT	(122)

More recently, alternative approaches to MELK targeting have focused on mechanisms beyond conventional kinase inhibition, including targeted protein degradation. The MELK-targeting PROTAC MGP-39 induced rapid MELK degradation, accompanied by suppression of mTOR signaling, cell-cycle arrest, and apoptosis. Compared with MELK-8a/JNJ-47117096, MGP-39 showed greater anticancer activity and lower cytotoxicity in the tested models, highlighting targeted protein degradation as a potential alternative to conventional MELK inhibition (126). These approaches broaden the therapeutic landscape of MELK targeting and suggest that alternative modes of target modulation may improve therapeutic efficacy and specificity.

### 5.3 Combination therapeutic strategies targeting MELK

The therapeutic efficacy of MELK inhibition is increasingly recognized to be enhanced in combination settings, reflecting MELK’s central role in regulating cell-cycle progression, transcriptional programs, stemness, and therapy resistance. As a result, combinatorial strategies have been explored across multiple therapeutic modalities. Targeting cell-cycle regulators represents one of the most effective strategies. Combined inhibition of MELK and CDKs results in pronounced anti-tumor effects, including disruption of FOXM1- and Wnt/β-catenin–dependent signaling, induction of G2/M arrest, and enhanced apoptosis, leading to significant tumor regression in preclinical models (127). MELK inhibition may also enhance responsiveness to endocrine therapy. In ERα-positive breast cancer, particularly ERα-positive/PR-positive/HER2-negative models, MELK inhibition promotes post-translational ERα degradation and reduces E2-induced ERα S118 phosphorylation and transcriptional activity, thereby suppressing ERα-dependent proliferation. Moreover, MELK inhibition exhibits synergistic antiproliferative effects with 4-hydroxytamoxifen, suggesting a potential strategy for enhancing endocrine therapy responses (128).

Beyond cytotoxic approaches, MELK inhibition has been shown to enhance responses to immunotherapy. In cervical cancer, MELK overexpression activates NF-κB signaling and promotes IL-6 secretion by tumor cells, thereby driving a shift in the Th1/Th2 balance toward a Th2-predominant phenotype and impairing cytotoxic CD8^+^ T-cell antitumor activity. IL-6 neutralization abrogated the effect of MELK overexpression on Th1/Th2 polarization, supporting an IL-6-dependent mechanism. Consistent with this immunomodulatory role, MELK overexpression conferred resistance to PD-1 blockade, whereas pharmacological MELK inhibition with OTSSP167 significantly enhanced the antitumor efficacy of PD-1 blockade in cervical cancer models. These findings provide a mechanistic rationale for combining MELK-targeted therapy with immune checkpoint inhibition (129). Additionally, MELK inhibition sensitizes cancer cells to therapeutic stress by impairing DDR and disrupting cell-cycle checkpoints, thereby increasing susceptibility to radiotherapy and targeted treatments (86, 130). DDR pathway status may therefore represent a potential stratification factor for these combinations, although its predictive value remains to be established. These findings support rational combination strategies in which MELK inhibition is paired with therapies targeting complementary vulnerabilities in cell-cycle regulation, hormone signaling, immune suppression, or DNA-damage responses.

### 5.4 Translational challenges in MELK-targeted therapy

Despite the therapeutic potential of MELK-targeted strategies, several challenges remain in translating these findings into clinically effective therapies. One major concern is the therapeutic window, as MELK is also expressed in normal proliferative compartments, including hematopoietic and germline cell lineages (26, 84, 116), raising the possibility of on-target effects during systemic inhibition. Consistent with this concern, OTSSP167 induced DNA double-strand breaks and follicular atresia in murine oocytes, although the clinical relevance of these findings remains unclear (9). In contrast, other preclinical studies have reported relatively limited effects of MELK inhibition on bone marrow compartments, suggesting that toxicity may vary across normal tissues and experimental setting (9). Another important challenge is patient selection. MELK expression alone may not reliably identify tumors that are functionally dependent on MELK, as pharmacological and genetic studies have demonstrated variable responses across cancer models (120, 131). Thus, MELK is unlikely to represent a universally essential therapeutic target and may instead constitute a context-dependent vulnerability. Future clinical development should therefore integrate tissue-specific safety assessment with functional dependency biomarkers and rigorous genetic and pharmacological target validation, rather than relying on MELK expression alone to guide patient selection.

## 6 MELK functional controversy in cancer

The role of MELK in cancer remains controversial, particularly because its high expression does not consistently translate into a requirement for tumor cell growth. Early RNA interference (RNAi)-based studies implicated MELK in cancer-cell proliferation and mitotic progression, with MELK depletion reported to impair growth in basal-like breast cancer models (9). However, this interpretation was challenged by CRISPR/Cas9-based studies showing that MELK loss had limited effects on cancer-cell proliferation and viability. Moreover, MELK expression strongly correlated with mitotic gene signatures, suggesting that its elevated expression may primarily reflect proliferative activity rather than an essential requirement for tumor growth (120, 131). Pharmacological evidence has further complicated the interpretation of MELK dependency. OTSSP167 retained antiproliferative activity in MELK-deficient cells, indicating that its effects cannot be attributed solely to MELK inhibition and highlighting the importance of target selectivity when evaluating pharmacological dependency (120). Thus, sensitivity to OTSSP167 alone should not be considered sufficient evidence of MELK dependence. Importantly, MELK dependency also appears to vary with cellular context and experimental conditions. In TNBC models, MELK depletion had limited effects under conventional high-density culture conditions but markedly impaired clonogenic growth at low seeding density. Similar effects were observed with both RNAi and CRISPR-mediated depletion, indicating that the discrepancy cannot be explained simply by the genetic approach. Compared with essential mitotic kinases such as PLK1 and AURKB, MELK therefore displayed a more conditional pattern of dependency (132). However, this conditional dependency is not uniformly observed. A separate CRISPR-based study reported no significant defect in clonogenic or anchorage-independent growth following MELK loss, further indicating that genetic background and experimental conditions may influence the observed phenotype (131). These findings suggest that differences across studies may arise from the biological context in which MELK is perturbed rather than from a simple distinction between RNAi and CRISPR approaches. Despite limited effects on proliferation under some conditions, MELK has also been implicated in other tumor-associated processes, including invasion, metastasis, therapeutic resistance, and tumor–immune interactions across different malignancies (18, 67, 130, 133). Thus, its biological relevance may extend beyond direct control of proliferation and depend on specific cellular states or tumor contexts. Overall, current evidence does not support MELK as a universally essential mitotic kinase in cancer. Instead, its expression appears to reflect proliferative activity, while its functional importance varies across cellular and tumor contexts. The observation that MELK dependency can emerge under specific growth conditions, together with the continued activity of OTSSP167 after MELK loss, highlights the need to evaluate MELK dependency in a context-specific manner and to carefully validate pharmacological effects. Thus, MELK expression alone may not be sufficient to identify tumors that will benefit from MELK-targeted therapy, and further work is needed to define the settings in which MELK provides a genuine growth or survival advantage.

## 7 Discussion

MELK extends beyond a simple mitotic regulator and is increasingly recognized as a context-dependent regulator of cellular adaptation, rather than a universal oncogenic driver (134). The evolutionary conservation of its modular architecture, specifically the unique spatial orientation of the N-terminal kinase domain relative to the UBA and KA1 domains, suggests a sophisticated mechanism of auto-inhibition and substrate targeting that remains under-explored (35, 135). While MELK has been widely recognized as a proliferation-associated kinase that is strongly linked to mitotic progression and tumor growth, emerging evidence indicates that its biological significance extends to adaptive oncogenic programs involving transcriptional, epigenetic, and survival pathways (49, 136). For instance, previous research demonstrated MELK-mediated FOXM1 activation links its mitotic functions with broader transcriptional programs involved in tumor progression (9). Similarly, another study suggested that MELK supports CSC properties and tumor initiation, highlighting its broader role beyond canonical cell-cycle regulation (12, 137). These observations position MELK as a signaling integrator that coordinates mitotic control with transcriptional and epigenetic programs underlying tumor progression.

A notable conceptual framework emerging from recent studies is the context-dependent functional inversion of MELK activity. Under physiological states, MELK functions within a pro-apoptotic framework, often acting as a scaffold for p53 stabilization and activation of stress-response pathways (23, 65). However, the malignant transformation process induces a functional inversion, in which MELK is recruited into pro-survival signaling networks, promoting activation of the NF-κB and AKT/mTOR pathways while attenuating tumor-suppressive responses (15, 16, 138). This shift represents a critical divergence point in tumor evolution, enabling cancer cells to transition from homeostatic growth to an aggressive, self-sustaining proliferative state. Consistent with this model, MELK also contributes to cancer stemness through the MELK-EZH2 axis (see Section 3.4), promoting epigenetic reprogramming and maintenance of stem-like tumor cell populations (139, 140). Additionally, it drives EMT by regulating transcription factors such as snail and slug, thereby enhancing tumor invasiveness and metastatic potential (141, 142). These multifaceted roles further support the notion that MELK functions as a regulator of tumor plasticity rather than as a single mitotic kinase.

Despite these insights, the functional necessity of MELK in cancer remains controversial. Early RNA interference-based studies suggested that MELK is essential for tumor cell survival, with its depletion impairing mitotic progression and inducing apoptosis in aggressive cancer cells (133, 143, 144). However, this view was challenged by Lin et al., who demonstrated using a CRISPR/Cas9-mediated knockout model that MELK is dispensable for cancer cell proliferation in several contexts, indicating that its role may not be universally required (117, 131). These findings suggest that MELK dependency is shaped by experimental and cellular context, with functional redundancy within mitotic signaling networks potentially contributing to this variability.

Pharmacological studies have further complicated the understanding of MELK function. Although several MELK inhibitors have been developed, their clinical translation remains limited (145). Among these, OTSSP167 has advanced to early-phase clinical trials (130), demonstrating potent anticancer activity across multiple tumor models, including suppression of proliferation, induction of apoptosis, and reduction of tumor growth (146). However, subsequent studies have shown that OTSSP167 exhibits limited selectivity and can inhibit multiple kinases, retaining activity even in MELK-deficient cells (116, 120). These findings suggest that the observed therapeutic effects may not be mediated exclusively by MELK inhibition. Consequently, the interpretation of pharmacological effects remains challenging, emphasizing the necessity of more selective inhibitors and rigorous target validation to distinguish MELK-specific activity from off-target effects.

From a translational perspective, these insights necessitate a shift in how MELK is conceptualized as a therapeutic target. The lack of universal dependency implies that MELK inhibition is unlikely to be broadly effective as monotherapy. Instead, its targeting may be most beneficial in combination strategies or in molecularly defined tumor subsets where MELK-driven pathways are selectively engaged. Moving forward, several key directions are essential to resolve the current ambiguity surrounding MELK biology. These include the development of next-generation selective inhibitors, integration of CRISPR-based functional genomics with single-cell analyses to map context-specific dependencies, and identification of predictive biomarkers for patient stratification. At the same time, the role of MELK within the tumor microenvironment remains poorly understood, particularly with respect to immune modulation and checkpoint signaling. In addition, exploration of synthetic lethal interactions involving MELK-associated pathways may provide new opportunities for targeted therapy. Importantly, this review advances the current knowledge by integrating MELK’s diverse and often conflicting roles into a unified conceptual framework and identifying context-dependent functional reprogramming as a central feature underlying its contribution to cancer progression and therapeutic relevance.

## 8 Conclusion

MELK is increasingly recognized as a context-dependent regulator whose function is reshaped during cancer progression. Rather than acting as a fixed component of mitotic control, MELK regulates within interconnected proliferative, stress-responsive, and adaptive signaling networks. This perspective accounts for the apparent disconnect between its strong clinical associations and its variable genetic dependency. Consequently, MELK represents a clinically relevant biomarker and a strategic component of combination-based therapeutic approaches, particularly in tumors exhibiting MELK-associated signaling dependencies. Identifying these context-specific dependencies will be critical to advancing the integration of MELK into diagnostic and precision oncology targets.

## Glossary

AKT: Protein kinase B
ALDH1: Aldehyde dehydrogenase 1
AMPK: AMP-activated protein kinase
ATM: Ataxia telangiectasia mutated
AURKB: Aurora kinase B
APC/C: Anaphase-promoting complex/cyclosome
BBB: Blood Brain Barrier
CCNB1: Cyclin B1
BRAF: v-Raf murine sarcoma viral oncogene homolog B
CDK1: Cyclin-dependent kinase 1
CCNB2: Cyclin B2
CRC: Colorectal cancer
DDR: DNA damage response
DLAT: Dihydrolipoamide S-acetyltransferase
EMT: Epithelial–mesenchymal transition
EZH2: Enhancer of zeste homolog 2
FAK: Focal adhesion kinase
FOXM1: Forkhead box protein M1
GC: Gastric cancer
HCC: Hepatocellular carcinoma
IV: intravenous
lncRNA: Long non-coding RNA
LUAD: Lung adenocarcinoma
MAPK: Mitogen-activated protein kinase
MCL-1: Myeloid cell leukemia 1
MELK: Maternal embryonic leucine zipper kinase
miRNA: MicroRNA
mTOR: Mechanistic target of rapamycin
NF-κB: Nuclear factor kappa B
pCR: Pathological complete response
PLK1: Polo-like kinase 1
PRAS40: Proline-rich AKT substrate of 40 kDa
RCC: Renal cell carcinoma
RTK: Receptor tyrosine kinase
SCLC: Small cell lung cancer
SQSTM1/p62: Sequestosome 1
SAC: Spindle assembly checkpoint
TCGA: The Cancer Genome Atlas
Trx: Thioredoxin
TOP2A: DNA topoisomerase II alpha
TNBC: Triple-negative breast cancer
TOPK: T-LAK cell-originated protein kinase
UBE2C: ubiquitin-conjugating enzyme E2 C
UBA: ubiquitin-associated
